# Terahertz-driven polymerization of resists in nanoantennas

**DOI:** 10.1038/s41598-018-26214-w

**Published:** 2018-05-17

**Authors:** Woongkyu Park, Youjin Lee, Taehee Kang, Jeeyoon Jeong, Dai-Sik Kim

**Affiliations:** Department of Physics and Astronomy and Center for Atom Scale Electromagnetism, Seoul National University, Seoul, 151-747 Korea

## Abstract

Plasmon-mediated polymerization has been intensively studied for various applications including nanolithography, near-field mapping, and selective functionalization. However, these studies have been limited from the near-infrared to the ultraviolet regime. Here, we report a resist polymerization using intense terahertz pulses and various nanoantennas. The resist is polymerized near the nanoantennas, where giant field enhancement occurs. We experimentally show that the physical origin of the cross-linking is a terahertz electron emission from the nanoantenna, rather than multiphoton absorption. Our work extends nano-photochemistry into the terahertz frequencies.

## Introduction

Localized surface plasmons in nanostructure can promote chemical reactions in a confined area below the diffraction limit via optical near-field enhancement, heat generation, and excitation of hot electrons. It increases the yield of chemical reactions and enables nanoscale spatial control. Therefore, many researchers in photocatalysis^1^ and photovoltaics^2^ have studied plasmon-induced chemistry. In particular, plasmon-induced (de)polymerization has been extensively studied due to its potential applications of nanolithography^3–6^, near-field mapping^7–13^, modulation^14^, and selective functionalization^15^.

However, the wavelength range of light used in previous experiments has been limited from the ultraviolet to near-infrared region. Despite the high potential applicability, there has been no report of resist polymerization using longer wavelength light partly because the photon energy required for the cross-linking of the resist is in the ultraviolet region. In order to induce resist polymerization in the visible and near-infrared regions where the photon energy is relatively small, an intense light source such as femtosecond laser or high field enhancement of the plasmonic system should be used to induce multiphoton absorption. In the longer wavelength region, such as mid-infrared, the number of photons required for cross-linking increases, so the probability of cross-linking decreases dramatically.

Recently, Richard G. Hobbs *et al*. reported that resist (de)polymerization can be induced by photoemission and hot-electron emission, rather than multiphoton absorption^16^. In addition, Simon L. Range *et al*., reported that terahertz electron emission can also induce resist depolymerization in microscale^17^. These experiments showed the possibility of resist polymerization in a new way. Nevertheless, nanoscale resist polymerization has not yet been demonstrated in the longer wavelength region.

Meanwhile, as the intense terahertz (THz) source develops, many kinds of research related to electron tunneling such as THz-scanning tunneling microscopy^18^, photoemission^19–23^, quantum plasmonics^24,25^, electromigration^26^, photon-assisted tunneling^27^, luminescence^28^ have been carried out. However, the realization of nanoscale chemical reactions using terahertz waves and tunneling electrons has not been reported yet. Then the following question arises: can we induce a nanoscale chemical reaction using field emission electron accelerated by the THz field?

**Figure 1:**
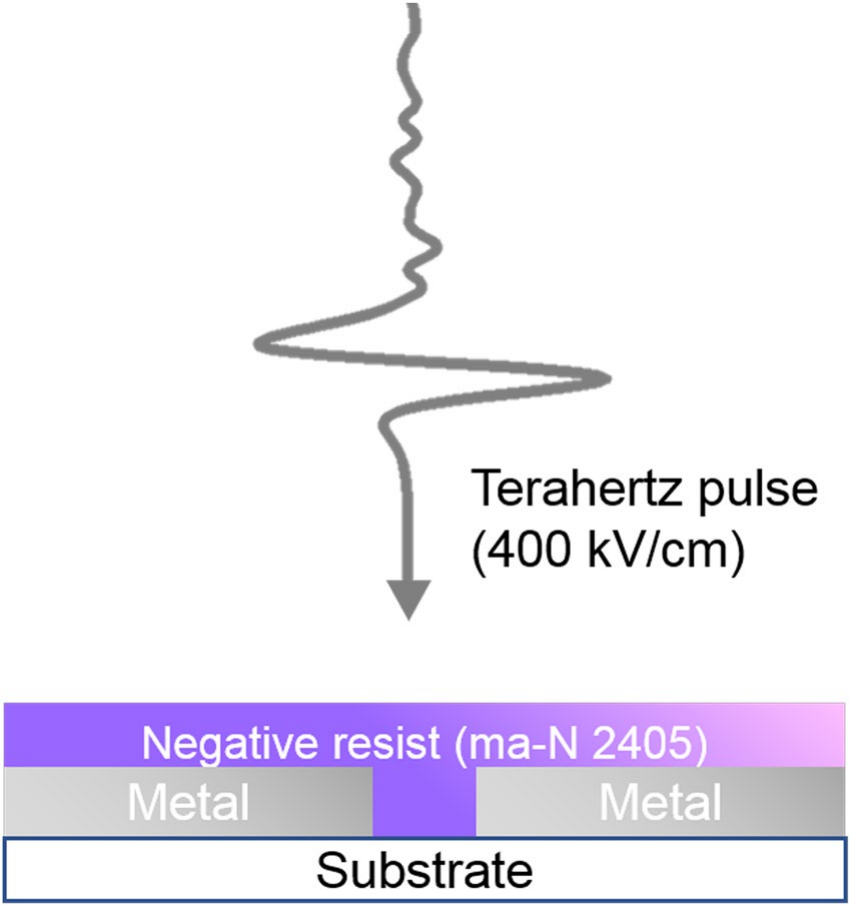
Schematics of terahertz illumination to photoresist/nanoantenna structure.

Here, we report nanoscale resist polymerization using intense THz pulse. Our strategy is to fabricate metal nano-slot antenna, which leads to strong field confinement and enhancement^29,30^. We spin-coat a resist onto the antenna and illuminate intense THz pulse to the resist-antenna structure (Fig. 1). To fabricate nanoantennas, we deposit 200-nm-thick Ag films on a 500-μm-thick quartz substrate. Then we pattern slot antenna arrays of length 60 μm, period 120 μm, and width 250 nm by focused ion beam (FIB). A square-shaped slot antenna array, which will be discussed later, is fabricated with the same method.

A negative tone photoresist ma-N 2405 (MicroChem), which is sensitive to deep-ultraviolet light and electron beam irradiation, is spin-coated on the nanoantenna sample (4000 rpm, 1 min). The resist thickness is about 500 nm, which is sufficient to cover the entire nanoantenna. The sample is soft-baked for 150 s at 90 °C. The ma-N 2405 resist consists of phenolic resin (novolak) and aromatic bisazide. Each is a polymeric bonding agent and a photoactive compound. This resist is not chemically amplified^31^. After THz illumination, the resist is developed by ma-D 525 (Microchem), which is composed of tetramethylammonium hydroxide in aqueous solution.

For the intense THz light illumination, we use an amplifier-based THz system. The THz light is generated via pulse-front-tilted optical rectification of a prism-cut lithium niobate (LiNbO_3_) crystal using femtosecond pulses from a Ti: sapphire regenerative amplifier (repetition rate: 1 KHz). The maximum electric field is 400 kV/cm at the focal point in the air. The peak frequency of the generated terahertz waves is 0.8 THz. The incoming THz wave illuminates the sample side first.

**Figure 2:**
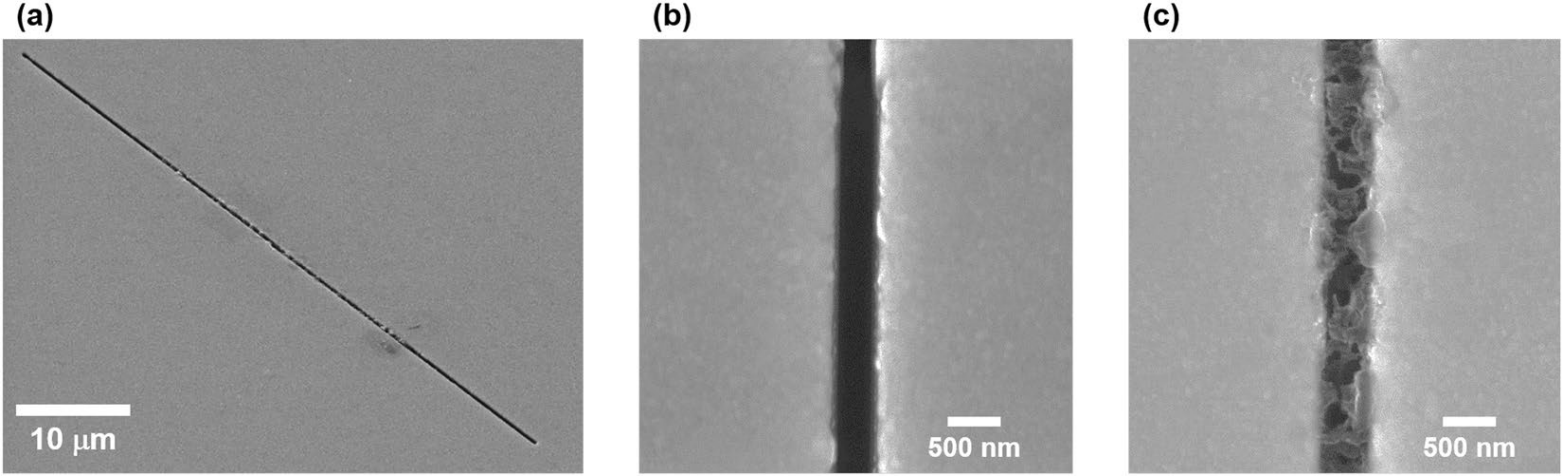
Terahertz-driven polymerization in a nano-slot antenna. (**a**) Cross-linked resist is positioned in the nano-slot antenna. (**b**) Nano-slot antenna before the resist coating & cross-linking. (**c**) Nano-slot antenna after the resist cross-linking.

We illuminate intense THz pulses to resist/antenna structure for 2 hours. The polarization direction of THz wave is perpendicular to the long axis of the slot antenna. The experiment is carried out in dark ambient environment in order to prevent cross-linking of the resist by ultraviolet light. As a result, resist polymerization occur in the slot antenna, as shown in Fig. 2a. For parallel polarization, the resist polymerization does not occur. As can be seen in Fig. 2b,c, the resist cross-linked antennas are distinctly different from the antennas without resist. We cannot observe any electromigration effect. We confirm that the resist is cross-linked only inside the antenna. That is, local field enhancement is an important factor for THz-driven polymerization. However, the photon energy of a terahertz wave is several meV, which is much smaller than the photon energy of ultraviolet light. It is very unlikely to induce multiphoton polymerization using terahertz field enhancement. Therefore, we infer that the process of resist cross-linking is different. As mentioned before, various experiments have been reported that electrons can be emitted when a strong terahertz wave is an incident on a metal structure. Therefore, we have inferred that polymerization is caused by terahertz electron emission.

**Figure 3:**
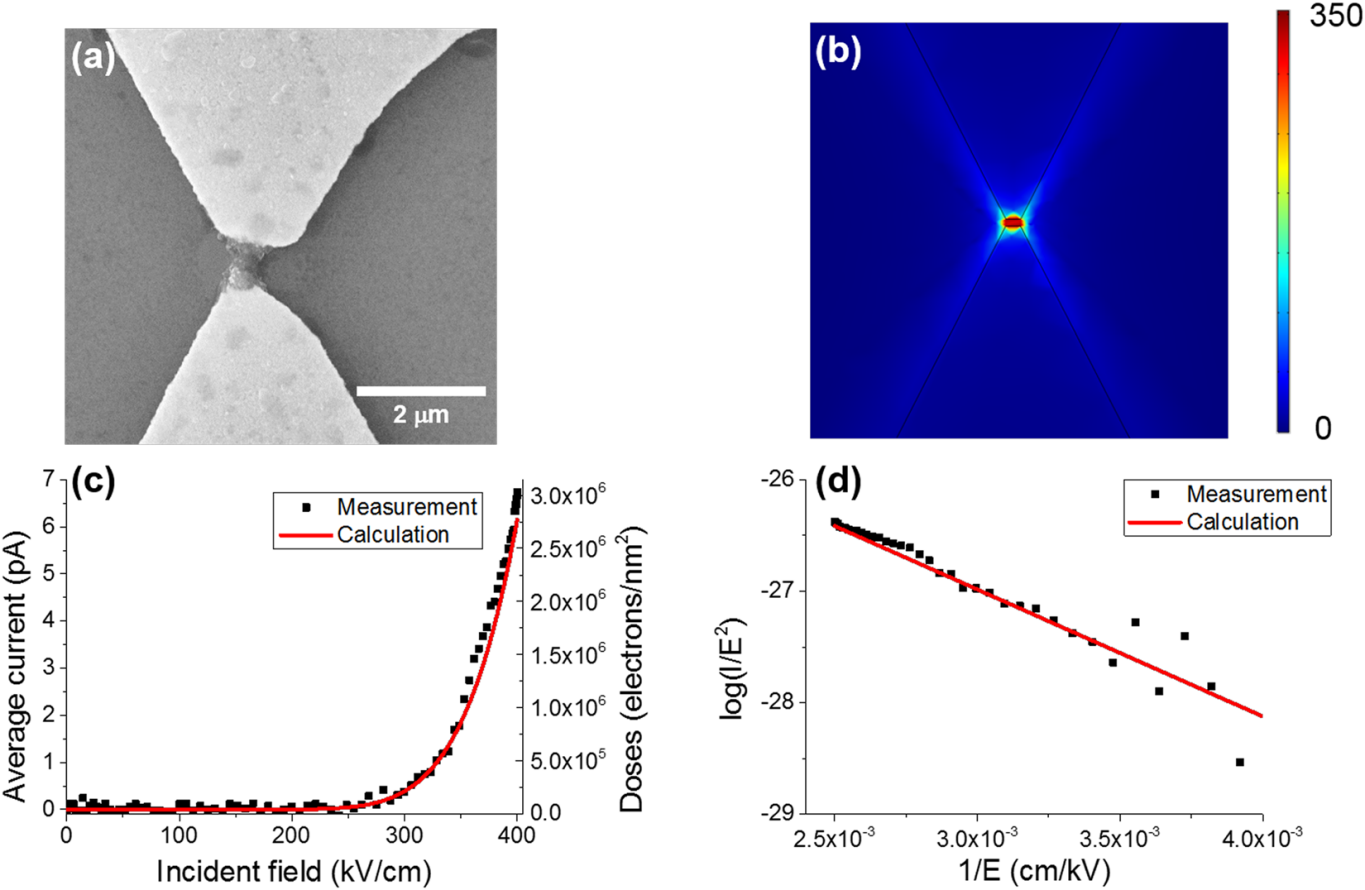
Terahertz-driven polymerization in a bowtie antenna. (**a**) Bowtie antenna after resists coating & cross-linking. (**b**) COMSOL simulations of the THz-resonant bowtie antenna. Field enhancement factor at the center of the antenna is about 300. (**c**) (Black dot) Measured electric current depending on the incident THz field amplitude. Electron emission starts at 250 kV/cm. (Red line) Calculated electric current depending on the incident THz field amplitude. Electron doses are calculated from the average electric current. (**d**) Fowler-Nordheim plot of the measured and calculated electric current.

To confirm our assumption, we fabricate terahertz-resonant bowtie nanoantennas. Bowtie nanoantennas are made by using electron beam lithography and ion milling. The period of the antennas is 360 μm, length of the long axis is 170 μm, length of the short axis is 85 μm, the gap size is 500 nm, respectively. Resist polymerization occurs again after 2-hour exposure to high-power terahertz waves. Cross-linking occurs only at the center of the bowtie antenna, where giant field enhancement occurs (Fig. 3a). To analyze the characteristics of the bowtie antenna, we perform finite element method simulation (COMSOL Multiphysics 5.3). The simulated electric field profile at the resonance frequency is shown in Fig. 3b. In this simulation, the gap size of the antenna is 1 μm. At the center of the bowtie antenna, the field enhancement factor reaches ~300. We measure the electric current while illuminating a terahertz wave to the sample to see directly if the electrons are emitted. Each pole of bowtie antennas is isolated, so electric current measurements can be made by using probe station^32^. In the measurement, we use 1 μm gap bowtie antenna, to match with simulation. The incoming terahertz wave illuminates the substrate side first. The intensity of the terahertz wave is adjusted using two wire grid polarizers. The polarization direction of the terahertz wave is not changed. We observe that the magnitude of the current exponentially increases as the amplitude of the THz wave increases (Fig. 3c, black dot). We analyze the data using Fowler-Nordheim plot and obtain a linear graph (Fig. 3d, black dot). When electrons are emitted from the metal by intense electric field, a current density follows Fowler-Nordheim equation^33^. The field emission current density *J* is given by the form^21^

$$J={\alpha }_{M}{\lambda }_{C}a{(\beta {E}_{0})}^{2}{{\rm{\Phi }}}^{-1}exp\{-\,\frac{vb{{\rm{\Phi }}}^{3/2}\,}{\beta {E}_{0}}\},$$

where *a* = 1.53 × 10^−6^ AeVV^−2^ and $b=6.829\times {10}^{9}\,{{\rm{eV}}}^{-\frac{3}{2}}{{\rm{Vm}}}^{-1}$, are first and second Fowler-Nordheim constants, respectively, *α*_*M*_ is the area efficiency of emission, *λ*_*C*_ is a characteristic supply correction factor, *v* is a correction factor associated with the barrier shape, Φ is the work function of the metal (in our case, the metal is gold.), *β* is the field enhancement factor, and *E*_0_ is the incident electric field. In our case, the barrier shape is triangular, so the factor α_M_, *λ*_*C*_, *v* becomes α_M_ = *λ*_*C*_ = *v* = 1^21^. We calculate theoretical electric current by using this equation and plot in Fig. 3c,d (red line). The calculation is consistent with the experimental data. In Fowler-Nordheim plot (Fig. 3d), the slope of the experimental data and theoretical data are almost identical. Considering the factor $vb{{\rm{\Phi }}}^{\frac{3}{2}}/\beta$, the slope is a function of field enhancement factor. That is, the field enhancement factor calculated by the COMSOL simulation is in good agreement with the experimental data. Therefore, we are able to confirm the terahertz photoemission directly through experiments, COMSOL simulation, and theoretical calculation. The electron emission occurs at the end of the bowtie antenna, which is similar to the hot electron emission at visible frequencies^34^. We also examine whether the amount of electrons emitted from the metals is sufficient to induce the resist polymerization. We obtain the electron doses from the measured and calculated electric current. In Fig. 3c, we plot electron doses as a function of the amplitude of the incident electric field. The exposure time is fixed as 2 hours. The calculated electron doses are about 10^5^~10^6^ electrons/nm^2^, which is much higher value comparing the one in the previous report using femtosecond laser pulse (10^4^~10^5^ electrons/nm^2^)^16^. We, therefore, conclude that the amount of electrons emitted by the terahertz wave is sufficient to induce the resist polymerization. Note that measurable field emission starts at 250 kV/cm. Considering the field enhancement factor 300, near-field reaches at 7.5 V/nm, which is much larger value than the threshold of 1 V/nm in the previous report by Jingdi Zhang *et al*.^22^. This is due to the difference between vacuum and atmospheric pressure. In atmospheric pressure, measurable field emission threshold becomes much higher than that in vacuum^35,36^. It may be because the electron transport between two metals can be affected by collision with molecules in the air^36^. In the ref.^35^, field emission threshold of atmospheric pressure becomes 7.5 times higher than that of vacuum. In our case, the threshold is also 7.5 times higher than that in the vacuum, which is similar to the ref.^35^.

**Figure 4:**
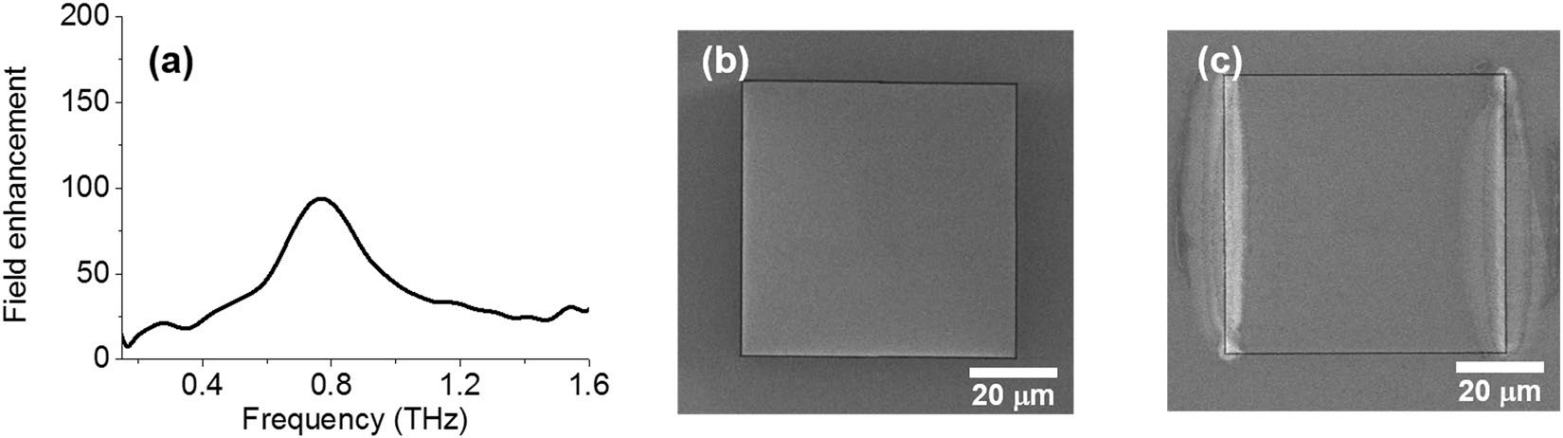
Possible applications of terahertz-driven polymerization. (**a**) Measured field enhancement spectra of the square-shaped nano-slot antenna. Field enhancement factor reaches about 100 at 0.8 THz. (**b**) SEM images of the square-shaped nano-slot antenna. (**c**) SEM image of resist/nano-slot antenna structure after THz exposure for 4 hours. The shape of cross-linked resists is similar to the profile of THz near-field, meaning that the cross-linked resists enable spatial photoemission mapping.

We also conduct a resist polymerization experiment using a square-shaped slot antenna to investigate the applicability of terahertz-driven polymerization. The antenna is fabricated by using FIB, the side length of the antenna is 60 μm, and the width of the gap is 400 nm. The antenna is made on a glass substrate. First, in order to investigate the characteristics of the square-shaped slot antenna, terahertz time-domain spectroscopy (THz-TDS) is performed. We perform THz-TDS using an oscillator-based system. A femtosecond Ti: sapphire laser with 780 nm center wavelength, 80 MHz repetition rate, and 130 fs pulse width (Mira 900 and Verdi V5, Coherent) is divided into pump and probe beam. Then pump beam and a biased GaAs emitter are used to generate single-cycle THz pulses. The pulses are focused with off-axis parabolic mirrors (NA = 0. 25) and incident normally in the sample. Transmitted THz wave is collected by parabolic mirrors (NA = 0.32) and detected by using (110)-oriented ZnTe crystal via electro-optic sampling. In this experiment, the incoming THz wave illuminates the substrate side first. Although the direction of the incident THz wave has changed, the transmission characteristics do not change, as previously reported^37^. The transmitted signal from the sample is normalized to the signal from the substrate. The normalized amplitude is about 0.03 at resonance and frequency is 0.8 THz. We determine the field enhancement factor using experimental values and Kirchhoff integral formalism^38^. Field enhancement factor is about 100 at 0.8 THz (Fig. 4a). We spin-coat resists on square-shaped slot antennas and expose intense terahertz pulses for 4 hours. After development, we can observe cross-linked resist not only inside the slot antenna but also outside. In addition, resist polymerization is observed only on the side perpendicular to the polarization direction of the terahertz waves. A comparison of Fig. 4b,c clearly shows the difference. The profile of cross-linked resist is similar to the near-field distribution in the terahertz nano-slot antenna^39^. Since photoemission is closely related to the local field enhancement factor, we can say that this is a spatial mapping of the photoemission. Note that resist polymerization occurs only inside the nano-slot antenna for 2-hour exposure time (Fig. 2c). For the sample in Fig. 4b, the 2-hour exposed resist is also polymerized only inside the nano-slot antenna. That is, by controlling the exposure time, we are able to perform not only a nanoscale chemistry and but also a photoemission mapping. To summarize, we are able to induce nanoscale chemical reactions using tunneling electrons generated by intense terahertz waves, and, show its potential application – photoemission mapping.

In conclusion, we demonstrate THz-driven resist polymerization using nanoantennas. The resist is cross-linked near the area where local field enhancement occurs in the nanoantenna. The enhanced THz waves in the nanoantenna induce photoemission and the electrons ejected from the metal cause the polymerization of the resist. Electric current measurement shows that the origin of cross-linking is photoemission, not multiphoton absorption. Our data shows remarkable results opening new possibilities for approaching nano-photochemistry with terahertz optics. Our efforts combining terahertz optics and nanochemistry can present great potential for nanogap-enhanced spectroscopy, nanolithography, near-field mapping, and modulation, etc.
